# Genetic Variation in *DROSHA* 3’UTR Regulated by hsa-miR-27b Is Associated with Bladder Cancer Risk

**DOI:** 10.1371/journal.pone.0081524

**Published:** 2013-11-28

**Authors:** Lin Yuan, Haiyan Chu, Meilin Wang, Xiaojian Gu, Danni Shi, Lan Ma, Dongyan Zhong, Mulong Du, Pu Li, Na Tong, Guangbo Fu, Chao Qin, Changjun Yin, Zhengdong Zhang

**Affiliations:** 1 Department of Urology, Jiangsu Province Hospital of TCM, Nanjing, China; 2 State Key Laboratory of Reproductive Medicine, Institute of Toxicology, Nanjing Medical University, Nanjing, China; 3 Department of Environmental Genomics, Jiangsu Key Laboratory of Cancer Biomarkers, Prevention and Treatment, Cancer Center, Nanjing Medical University, Nanjing, China; 4 Department of Genetic Toxicology, the Key Laboratory of Modern Toxicology of Ministry of Education, School of Public Health, Nanjing Medical University, Nanjing, China; 5 Department of Urology, the First Affiliated Hospital of Nanjing Medical University, Nanjing, China; 6 Department of Urology, the Huai-An First Affiliated Hospital of Nanjing Medical University, Huai-An, China; Geisel School of Medicine at Dartmouth, United States of America

## Abstract

**Purpose:**

miRNAs can regulate the biological processes, including differentiation, proliferation and apoptosis. DICER and DROSHA are two members of RNase III family, playing pivotal roles in the pathway of miRNAs biogenesis. In this study, we hypothesized that genetic variations of the DICER and DROSHA genes were associated with the bladder cancer risk.

**Experimental Design:**

We performed a case-control study of 685 bladder cancer cases and 730 controls to investigate the association between the seven functional SNPs of *DICER* and *DROSHA* genes and bladder cancer risk. We then evaluated the functionality of the important SNPs.

**Results:**

We found that rs10719T>C polymorphism located in 3’ untranslated region (UTR) of *DROSHA* gene was associated with the increased risk of bladder cancer. Stratified analysis suggested that rs10719TC/CC genotype can increase risk of bladder cancer among male patients (Adjusted OR = 1.34, 95% CI = 1.05-1.70, *P* = 0.018), and ever smokers (1.56, 1.14-2.14, 0.006), compared with TT genotype. Furthermore, *DROSHA* rs10719T>C polymorphism was predicted to regulate the binding activity of hsa-miR-27a/b. Luciferase reported gene assay confirmed that rs10719 T to G substitution disrupted the binding site for hsa-miR-27b, resulting the increased levels of DROSHA protein.

**Conclusions:**

Taken together, these findings suggested that *DROSHA* rs10719T>C polymorphism may be associated with bladder cancer risk in a Chinese population, and hsa-miR-27b can influence the expression of DROSHA protein by binding with 3’UTR.

## Introduction

Bladder cancer accounts for approximately 2% of all human malignancies with an estimated 72,570 new cases and 15,210 deaths in the USA in 2013 alone [1]. In China, the overall registered bladder cancer incidence was 7.49/100,000 in 2008, and the incidence of bladder cancer was rising during 1998-2008 (average growth rate per year, 4.60%) [2]. More than 90% of the bladder cancer is transitional cell carcinoma. The incidence of bladder cancer is generally high in the USA and Europe, but low in Asia. Like other common cancers, bladder cancer is a complex disease caused by both genetic and environmental risk factors. Cigarettes smoking, occupational and environmental exposures are well-established known risk factors for bladder cancer [3]. It has been reported that FGFR3 mutation was associated with the low bladder tumor grade and stage, and the mutations of TP53 and FGFR3 showed an inverse relationship [4-6]. Recently, several genome-wide association studies (GWAS) with replications have identified that the common genetic variations are associated with susceptibility to bladder cancer [7-11]. Tang et al. also identified that an uncommon coding variant of *UGT1A* locus (GWAS related) can affect *UGT1A* mRNA expression and decrease the risk of bladder cancer [12], However, the exact mechanisms of the bladder cancer did not be clarified.

MicroRNAs (miRNAs) are a class of small non-coding RNA molecules of ~22 nucleotides, which regulate gene expression at the post-transcriptional level through binding the 3’ untranslated region (UTR) of target genes mRNA [13]. miRNAs are generated in a two-stepwise processing pathway mediated by two major enzymes (DICER and DROSHA): In the nucleus, longer precursors are processed into primary RNAs (pri-miRNAs) by the RNase II and then pri-miRNAs are processed by the RNase enzyme (DROSHA) into precursors (pre-miRNAs) with a stem-loop structure [14,15]. The pre-miRNAs are exported from the nucleus to the cytoplasm by the exportin-5 protein. In the cytoplasm, pre-miRNAs are processed into mature miRNAs by another RNase enzyme (DICER). The mature miRNAs play roles by incorporating into the RNA-induced silencing complex (RISC) [16]. It has been suggested that miRNAs are predicted to regulate 30% of human genes [17]. Recently, several studies showed that miRNAs could act as oncogenes and tumor suppressors by targeting 3’UTR of important genes [18,19] and the genetic variants in 3’UTR of the miRNA target genes would affect miRNA-mediated gene regulation, eventually resulting the increased risk of cancer [19,20]. It is worth to note that DICER and DROSHA play the crucial role in carcinogenesis. Accumulated evidences have shown that imbalance *DICER* and *DROSHA* expression levels are associated with bladder cancer risk [21-23]. Recently, Han and his colleagues also found that *DICER* and *DROSHA* expression levels were up-regulated in bladder cancer tissues compared to the matched normal bladder tissues, and silencing DICER or DROSHA can inhibit cell proliferation and induce cell apoptosis [24]. Here, we propose that it is warranted to investigate the roles of the *DICER* and *DROSHA* in the susceptibility to bladder cancer.

Up to now, several studies have investigated the association between the genetic variants of the *DICER* and *DROSHA* genes and risk of diseases. Lin et al. reported that the *DICER* and *DROSHA* haplotypes were associated with the altered survival and recurrence of renal cell carcinoma patient in Caucasians [25]. However, the genetic variants of *DICER* and *DROSHA* were not associated with the development of renal cell carcinoma [26]. In addition, Yang et al. also observed the similar result of the bladder cancer in Caucasians [27]. Recently, Qin et al. showed that the *DICER* and *DROSHA* polymorphisms might modify the risk of abnormal semen parameters and be associated with the Chinese male infertility [28]. Taken together, we hypothesized that the genetic variants of *DICER* and *DROSHA* are also be associated with the susceptibility to bladder cancer in a Chinese population.

On the basis of this postulation, we selected seven polymorphisms of *DICER* (rs12323635CT, rs13078TA, rs1057035TC, and rs3742330AG) and *DROSHA* (rs2291109AT, rs10719TC, and rs642321CT) to evaluate the association between the genetic variants of *DICER* and *DROSHA* genes and risk of bladder cancer. In this study, we found that *DROSHA* 3’UTR polymorphism rs10719TC can increase the risk of bladder cancer in a Chinese population, which was located near a miRNA binding site. Furthermore, we conducted a series of functional assays on *DROSHA* 3’UTR polymorphisms to reveal its molecular mechanism. 

## Materials and Methods

### Study subjects

In the present study, we included 685 histopathologically confirmed bladder transitional cell carcinoma and 730 cancer-free controls. Included study subjects were recruited from the First Affiliated Hospital and Huai-An Affiliated Hospital of Nanjing Medical University, and Jiangsu Province Hospital of Traditional Chinese Medicine (TCM) between January 2003 and January 2010. The detailed method of recruiting study subjects for the study had been described previously [29]. Pathological diagnosis for bladder tumor stage was according to the 2002 International Union Against Cancer tumor–nodes–metastasis classiﬁcation and the World Health Organization 1973 grading of urothelial papilloma was used to define the bladder cancer grade: well differentiated (grade 1, G1), moderately differentiated (grade 2, G2) or poorly differentiated (grade 3, G3). Bladder cancer patients were excluded, if which had previous cancer, metastasized cancer from other origin, previous radiotherapy or chemotherapy. The cancer-free subjects were recruited from those who were seeking health care in the outpatient departments at the hospital. The cancer-free controls were matched by age (± 5 years) and sex to the cases, which were genetically unrelated to the cases and had no individual history of cancer including melanoma skin cancer. The cancer-free subjects who had symptoms suggestive of bladder cancer, such as hematuria, were excluded. We used a short questionnaire to obtain the demographic and risk factor information from the included subjects. In this study, we defined ever smokers (former and current smokers) based on smoking condition. Subjects who smoked daily for >1 year were defined as ever smokers. Ever smokers who had quit smoking for >1 year were defined as former smokers and the others as current smokers. This case-control study was approved by the institutional review board of Nanjing Medical University. All individuals signed informed consents, and each subject donated 5 ml blood sample for genomic DNA extraction.

### SNPs selecting and genotyping

In this study, we studied the genetic variants of *DICER* and *DROSHA* genes, which play the important roles in the miRNA biogenesis. Here, we focused on studying the single nucleotide polymorphisms (SNPs) spanning these two genes (HapMap Data Release 27), including 2 kb upstream and 2 kb downstream using the Haploview software [30]. The following criteria should be included: (i) SNPs should be located in the 5’ flanking region, 5’ UTR, 3’UTR, and coding region with amino acid changes, (ii) minor allele frequency (MAF) > 5% in Han Chinese in Beijing (CHB). According to the criteria, four SNPs were identified in *DICER* (rs12323635, rs13078, rs1057035, and rs3742330) and three SNPs in *DROSHA* (rs2291109, rs10719, and rs642321).

Genomic DNA was extracted from peripheral blood lymphocytes of the subject. The included seven SNPs were genotyped in all 1415 subjects using the MGB TaqMan probe Assay (7900HT Real Time PCR System, Applied Biosystems, Foster City, USA). About 10% of the samples were randomly selected for repeated genotyping for validation, and the results were 100% concordant. Genotype analysis was performed by two persons independently in a blinded fashion and controls were included in each plate to ensure accuracy of the genotyping. However, several samples failed in genotyping were due to DNA quality, and we would exclude them in the further analyses. Table 1 provided the primary information of the selected seven SNPs.

**Table 1:** Primary information of genotyped SNPs.

**Gene (accession no.) and locus**	**NCBI rs no.**	**Position** ^a^	**Location**	**Base change**	**MAF**			***P* for HWE** ^c^	**Genotyping rate (%)**
					**HapMap** ^b^	**Case**	**Controls**		
*DICER* (NM_030621) 14q31	rs12323635	95625711	promoter	C>T	0.333	0.376	0.378	0.067	98.2
	rs13078	95556747	3’UTR	T>A	0.056	0.057	0.061	0.131	99.1
	rs1057035	95554142	3’UTR	T>C	0.111	0.112	0.110	0.741	100.0
	rs3742330	95553362	3’UTR	A>G	0.267	0.331	0.332	0.257	99.6
*DROSHA* (NM_013235) 5p14-p13	rs2291109	31532301	promoter	A>T	0.211	0.219	0.234	0.062	100.0
	rs10719	31401447	3’UTR	T>C	0.233	0.282	0.243	0.437	99.7
	rs642321	31401003	3’UTR	C>T	0.467	0.474	0.505	0.655	100.0

^a^ SNP position in NCBI dbSNP (http://www.ncbi.nlm.nih.gov/SNP).

^b^ MAF from the HapMap databases (http://www.hapmap.org).

^c^ HWE *P* value in the control group.

### Bioinformatics analysis of *DROSHA* 3’UTR

**Figure 1:**
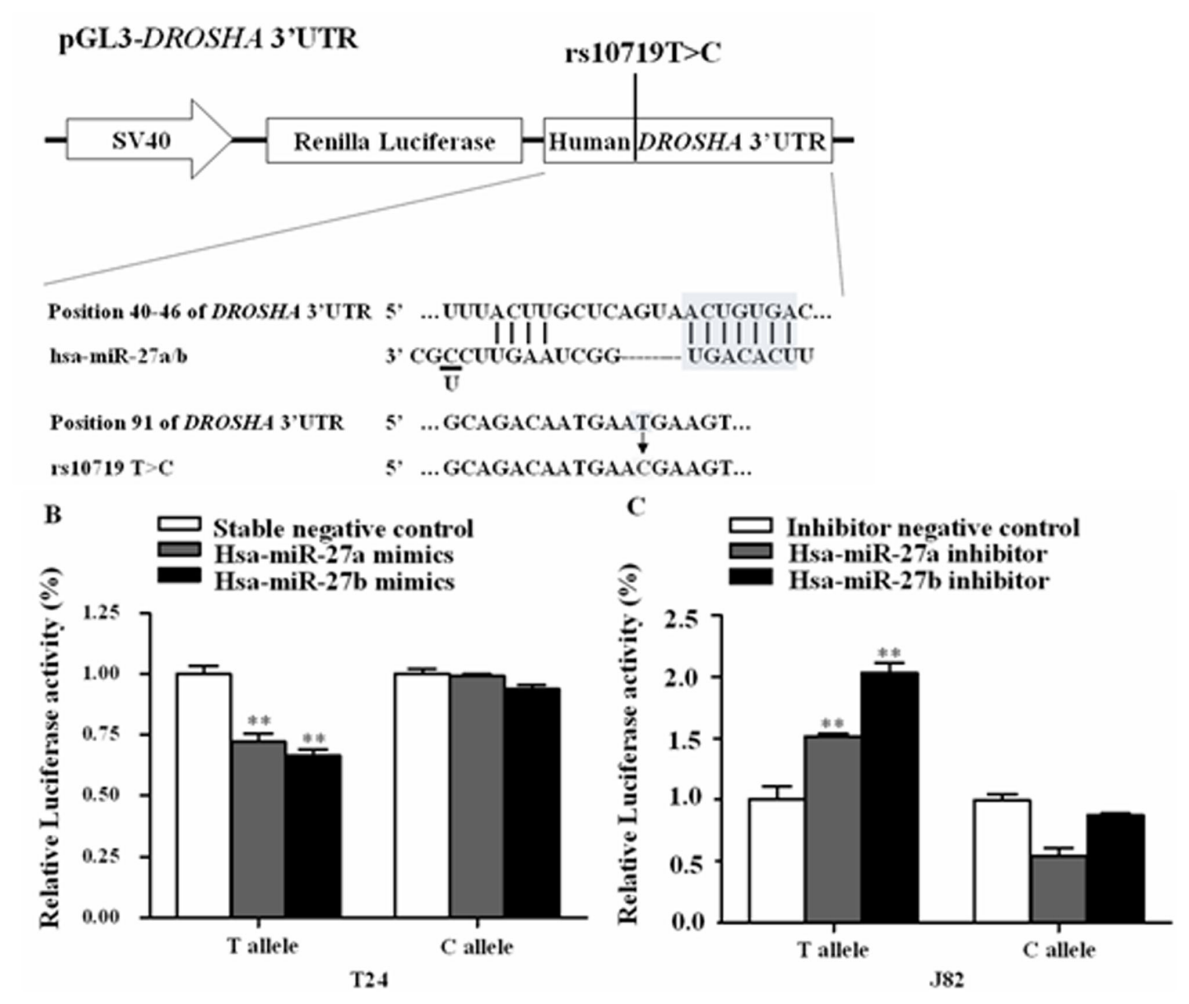
Characterization and functional analysis of the DROSHA 3’UTR. (A) DROSHA 3’UTR was predicted a binding site for hsa-miR-27a/b. The sequence of hsa-miR-27a and hsa-miR-27b only had one base difference (underlined). Scanning about ±100bp regions of the binding site, we only found that rs10719T>C was located in this region. Predict effect of allelic variation at rs10719 on hsa-miR-27a/b recognition and the construct of pGL3-DROSHA 3’UTR-T/C containing renilla luciferase gene and full-length 3’UTR of DROSHA gene with different alleles of rs10719 (arrow: T>C substitution). (B, C) Luciferase reporter assays to measure rs10719T or C allele difference with the presence or interference of hsa-miR-27a/b. In (B), T24 cells seeded on 24-well plates were transiently co-transfected with constructs and hsa-miR-27a/b mimics or stable negative control (NC). In (C), J82 cells seeded on 24-well plates were transiently co-transfected with constructs and hsa-miR-27a/b inhibitors or inhibitor NC. Results are shown as relative luciferase activity versus NC. Data were from three independent transfection experiments. _**,_ *P* < 0.05.

Based on bioinformatics analysis, we predicted that hsa-miR-27a/b can bind with the 3’UTR region of *DROSHA* by using four common websites (Target Scan: http://www.targetscan.org/, miRanda: http://www.microrna.org/, Microcosm: http://www.ebi.ac.uk/enright-srv/microcosm/cgi-bin/targets/v5/genome.pl, and PITA: http://genie.weizmann.ac.il/) (Figure 1A). We considered that the combination of these approaches would greatly reduce the possibility of false positive.

### Real-time quantitative reverse transcription-PCR (RT-PCR) assay

In order to evaluate the endogenous expression level of hsa-miR-27a/b, four bladder cancer cell lines (EJ, T24, J82, and 5637) seeded into 20 cm^2^ plates were subjected to extraction of the total RNA isolated from cells using Trizol Reagent (Invitrogen, CA, USA). Table S1 in File S1 showed the primers information of hsa-miR-27a/b and U6. The reverse transcriptase reactions (10 μl) contained 2 μl total RNA (500 ng/μl), 1 μl 10×AMV RT buffer, 10 pmol each of dNTPs (Toyobo, Tsuruga, Japan), 0.75 μl antisense looped primer mix, 0.25 U/μl RNase Inhibitor (Toyobo, Tsuruga, Japan), 1U/μl AMV reverse transcriptase. The mixture was incubated at 16 °C for 15 min, 42 °C for 60 min, and 85 °C for 5 min. Next, Applied Biosystems 7900HT Real Time PCR System was used to perform real-time quantification PCR (ABI, CA, USA) based on the SYBR-Green method (Toyobo, Tsuruga, Japan). All reactions were conducted in triplicate. Fold changes were normalized to the expression levels of U6.

For detection of the correlation between the *DROSHA* mRNA levels and rs10719 T>C polymorphism *in vivo*, a total of 61 bladder tumor tissues with different genotypes (32 for TT, 24 for TC, and 5 for CC genotypes) were subjected to extraction of the total RNA using Trizol Reagent (Invitrogen, CA, USA). Bladder tumor tissues were preserved in liquid nitrogen after being removed from the body. The total RNA was evaluated by both reverse transcriptase reaction and real-time quantitative PCR based on the SYBR-Green method (Toyobo, Tsuruga, Japan). The cDNA was used for the amplification of *DROSHA* gene and an endogenous control gene *GAPDH*. The primers information of *DROSHA* and *GAPDH* genes were included in the Table 1. Fold changes were normalized by the expression levels of *GAPDH* and each assay was performed in triplicate.

### *DROSHA* 3’UTR containing rs10719TC reporter gene constructs and Luciferase reporter assays

To construct the luciferase reporter plasmids of *DROSHA* 3’UTR, *DROSHA* 3’UTR fragments (937bp) carrying the major rs10719T allele were amplified by PCR. The primers were 5’-ACCTTGGTACCCCAGATGAGACTGAAGACATC-3’ (forward) and 5’-ACCTTCTCGAGGCACTCACTATATATTTGCTG-3’ (reverse). The PCR products were extracted and separated by agarose gel, which were cloned with TA cloning Kit (Invitrogen, CA, USA). In addition, the fragment containing minor rs10719C allele was conducted using the following primers: 5’-TAGTTTTCCTGCAGACAATGAACGAAGTGTGC-3’ (forward) and 5’-TTTATTTCAATGAGCACACTTCGTTCATTGTC-3’ (reverse). Finally, the amplified fragment carrying T or C allele was inserted downstream of the luciferase gene in a pGL3-promoter plasmid and then the plasmid containing T or C allele was conducted, which were confirmed by sequencing.

For luciferase reporter assay, T24 and J82 cells were placed in 24-well plates (1×10^5^ cells per well) and then cotransfected with pGL3-*DROSHA* 3’UTR-T or pGL3-*DROSHA* 3’UTR-C and pRL-SV40 (50:1). The mimics and inhibitors of hsa-miR-27a/b and their negative controls (GenePharma, Shanghai, China) were cotransfected with the reporter plasmids at a final concentration of 20nmol/μl. Forty-eight hours after transfection in T24 and J82 cells, luciferase activity in lysates was measured with a Dual-Luciferase Reporter Assay System (Promega, WI, USA) and normalized against the activity of the pRL-SV40. Assays were followed by the manufacture’s suggestions. Independent triplicate experiments were performed for each plasmid construct.

### Statistical analysis

The Hardy-Weinberg equilibrium of the genotype distribution among the controls was applied by using a goodness-of –fit χ^2^ test. The frequency distributions of selected demographic variables between the cases and controls were tested using χ^2^ test. Genotype-specific odds ratios (ORs) and their 95% confidence intervals (CIs) were calculated by unconditional univariate and multivariate logistic regression analyses. The multivariate adjustment included the age, sex and smoking status (never and ever smoking). In addition, Kruskal-Wallis one-way ANOVA tests were used for analyzing the results of *DROSHA* mRNA expression *in vivo*. In this study, the relative luciferase reporter gene expression for T or C allele was calculated separately. Student’s *t* test was used to evaluate the differences in the expression levels of luciferase reporter gene among subgroups. All tests were two-sided using the SAS software (version 9.1; SAS Institute, Inc, Cary, NC, USA) and *P* < 0.05 was considered statistically significant.

## Results

### Association study between the polymorphisms of *DICER* and *DROSHA* and bladder cancer risk

The characteristics of the 685 bladder transitional cell carcinoma patients and 730 controls are summarized in Table 2. Herein, we did not observe statistical difference in the distribution of age (*P* = 0.144) and sex (*P* = 0.825) between patients and controls. However, there were more ever smokers (55.6%) among the patients more than among the controls (38.4%), and this difference was statistically significant (*P* < 0.001). These variables were adjusted for the subsequent multivariate logistic regression analysis. Of the 685 cases, there were 315 tumor grade 1 (46.0%), 261 tumor grade 2 (38.1%) and 109 tumor grade 3 (15.9%) patients. In addition, 435 (63.5%) had superficial tumors and remaining 250 (36.5%) had invasive tumors.

**Table 2:** Frequency distributions of selected variables between the bladder cancer cases and cancer-free controls.

Variables	Cases (n = 685)			Controls (n = 730)		*P* ^a^
	N	%		N	%	
Age						
≤ 65	329	48.0		379	51.9	0.144
> 65	356	52.0		351	48.1	
Sex						
Male	554	80.9		587	80.4	0.825
Female	131	19.1		143	19.6	
Smoking status						
Never	304	44.4		450	61.6	< 0.001
Ever	381	55.6		280	38.4	
Former	172	25.1		72	9.9	
Current	209	30.5		208	28.5	
Tumor grade						
G1	315	46.0				
G2	261	38.1				
G3	109	15.9				
Tumor stage						
Superficial (pT_a_-pT_1_)	435	63.5				
Invasive (pT_2_-pT_4_)	250	36.5				

^a^ Two-sided χ^2^-test for the frequency distribution of selected variables between bladder cancer cases and cancer-free controls

The seven SNPs genotype frequencies among the control were in agreement with the Hardy-Weinberg equilibriums (*P* > 0.05; Table 1). As shown in Table 3, we observed that subjects with the *DROSHA* 3’UTR rs10719C allele (TC and CC genotypes) had a 1.24-fold increased risk of bladder cancer (Adjusted OR = 1.25, 95% CI = 1.01-1.55, *P* = 0.041) compared with the rs10719TT genotype. Meanwhile, we observed that the distribution of rs10719TC genotypes between the cases and controls showed significant difference (*P* = 0.017). However, we did not observe any significant differences in genotype distribution of the *DICER* rs12323635CT, rs13078TA, rs1057035TC, rs3742330AG and *DROSHA* rs2291109AT, rs642321CT polymorphisms between the cases and controls (all *P* >0.05, Table 3). We further evaluated the effect of included seven polymorphisms on bladder cancer risk by stratifying by sex and smoking status. As shown in Table S2 in File S1, we found that rs10719TC polymorphism can increase risk of bladder cancer among male patients (Adjusted OR = 1.34, 95% CI = 1.05-1.70, *P* = 0.018), and ever smokers (Adjusted OR = 1.56, 95% CI = 1.14-2.14, *P* = 0.006).

**Table 3:** Genotype frequencies of the *DICER* and *DROSHA* SNPs among bladder cancer cases and controls and their association with bladder cancer risk.

Genotypes	Cases			Controls		Crude OR (95% CI)	Adjusted OR (95% CI)^a^	*P* ^a^	*P* ^b^
	N	%		N	%				
*DICER*									
rs12323635CT	680			710					
CC	266	39.1		286	40.3	1.00 (reference)	1.00 (reference)		0.887
CT	321	47.2		311	43.8	1.11 (0.88-1.40)	1.09 (0.86-1.37)	0.485	
TT	93	13.7		113	15.9	0.89 (0.64-1.22)	0.88 (0.63-1.22)	0.441	
CT/TT	414	60.9		424	59.7	1.05 (0.85-1.20)	1.03 (0.83-1.28)	0.793	
rs13078TA	679			723					
TT	603	88.8		640	88.3	1.00 (reference)	1.00 (reference)		0.640
AT	75	11.1		78	10.8	1.02 (0.73-1.43)	0.99 (0.70-1.39)	0.937	
AA	1	0.2		5	0.7	0.21 (0.03-1.82)	0.30 (0.03-2.57)	0.271	
AT/AA	76	11.2		83	11.5	0.97 (0.70-1.35)	0.95 (0.68-1.33)	0.768	
rs1057035TC	685			730					
TT	548	80.0		577	79.0	1.00 (reference)	1.00 (reference)		0.857
TC	120	17.5		145	19.9	0.87 (0.67-1.14)	0.88 (0.67-1.16)	0.371	
CC	17	2.5		8	1.10	2.34 (0.96-5.23)	2.36 (1.00-5.59)	0.051	
TC/CC	137	20.0		153	21.0	0.94 (0.73-1.22)	0.96 (0.74-1.25)	0.753	
rs3742330AG	683			727					
AA	302	44.2		331	45.5	1.00 (reference)	1.00 (reference)		0.942
AG	310	45.4		309	42.5	1.10 (0.88-1.37)	1.09 (0.87-1.37)	0.437	
GG	71	10.4		87	12.0	0.90 (0.63-1.27)	0.88 (0.61-1.25)	0.464	
AG/GG	381	55.8		396	54.5	1.06 (0.86-1.30)	1.05 (0.84-1.30)	0.686	
*DROSHA*									
rs2291109AT	685			730					
AA	421	61.5		419	57.4	1.00 (reference)	1.00 (reference)		0.332
AT	228	33.3		280	38.4	0.81 (0.65-1.01)	0.79 (0.63-0.99)	0.044	
TT	36	5.3		31	4.3	1.16 (0.70-1.90)	1.20 (0.72-2.00)	0.482	
AT/TT	264	38.5		311	42.6	0.85 (0.68-1.05)	0.83 (0.67-1.03)	0.098	
rs10719TC	684			727					
TT	352	51.5		413	56.8	1.00 (reference)	1.00 (reference)		0.017
TC	278	40.6		275	37.8	1.19 (0.95-1.48)	1.20 (0.96-1.50)	0.116	
CC	54	7.9		39	5.4	1.63 (1.05-2.51)	1.61 (1.03-2.50)	0.036	
TC/CC	332	48.5		314	43.2	1.24 (1.01-1.53)	1.25 (1.01-1.55)	0.041	
rs642321CT	685			730					
CC	197	28.8		176	24.1	1.00 (reference)	1.00 (reference)		0.107
CT	326	47.6		371	50.8	0.79 (0.61-1.01)	0.79 (0.61-1.02)	0.075	
TT	162	23.7		183	25.1	0.79 (0.59-1.06)	0.78 (0.58-1.06)	0.112	
CT/TT	488	71.2		554	75.9	0.79 (0.62-1.00)	0.79 (0.62-1.01)	0.056	

^a^ Adjusted for age, sex, and smoking status (never and ever) in logistic regression model.

^b^ Two-sided chi-square test for distribution of allele frequency (minor allele versus major allele).

### *DROSHA* 3’UTR rs10719TC affects *DROSHA* expression by regulating the hsa-miR-27b binding

In order to explore the possible mechanism of the *DROSHA* 3’UTR in the bladder cancer risk, we performed the functional assays. Based on bioinformatics analysis, the *DROSHA* 3’UTR was predicted a binding site for hsa-miR-27a/b (Figure 1A). Here, we demonstrated that rs10719TC was located 46bp downstream of the hsa-miR-27a/b binding site in the *DROSHA* 3’UTR.

As shown in Figure S1 in File S1, real-time quantitative RT-PCR assay suggested that endogenous expression levels of hsa-miR-27a/b in J82 cell line were more significantly higher than other cells (T24, EJ, and 5637) (*P* < 0.001). Here, we chose J82 and T24 cell lines in the further luciferase assay. Then, we used the plasmids for transient co-transfection with the T24 cells [stable negative control (NC) miRNA: stable NC or hsa-miR-27a/b mimics] and J82 cells (inhibitor NC or hsa-miR-27a/b inhibitor). As shown in Figure 1B (T24 cell), hsa-miR-27a/b suppressed luciferase expression in the presence of rs10719T allele, comparing with NC (*P* < 0.05), but not the rs10719C allele (*P* > 0.05). Additionally, we also found that the inhibition of hsa-miR-27a/b expression can increase luciferase expression efficiently for the rs10719T-containing plasmid rather than the rs10719C-containing plasmid in J82 cells (*P* < 0.05; Figure 1C). However, we also found that a significant decrease in luciferase expression, when we added hsa-miR-27a inhibitor to allele C in J82 cells. The similar result did not be observed in adding hsa-miR-27b inhibitor to allele C in J82 cells. Maybe, hsa-miR-27a did not directly affect DROSHA luciferase expression. Our data suggested that rs10719 T to C substitution acts as a loss-of-function mutation and would affect DROSHA luciferase expression by hsa-miR-27b target.

In the present study, we also performed RT-PCR assay to explore whether hsa-miR-27b affected DROSHA expression by degrading mRNA or suppressing mRNA post-translational translation (Figure S1 in File S1). A total of 61 bladder tumor tissues with different genotypes of the *DROSHA* rs10719TC polymorphism were used to assess the expression of *DROSHA* mRNA. No significant difference levels of *DROSHA* mRNA among individuals with the TT, TC and CC genotypes was observed (*P* > 0.05). Taken together, hsa-miR-27b may affect DROSHA expression by regulating protein translation.

## Discussion

In the present study, we investigated the association between seven polymorphisms of *DICER* and *DROSHA* genes and bladder cancer risk in a Chinese population, and identified that rs10719TC polymorphism adjacent to the hsa-miR-27b binding site in *DROSHA* 3’UTR was associated with significantly increased the risk of bladder cancer. Functional assays indicated that *DROSHA* rs10719 T to C substitution can decrease the binding activity of hsa-miR-27b with *DROSHA* 3’UTR.

DROSHA is a member of RNase III superfamily and is an important nuclease that executes the initial step in miRNA processing by cutting pri-miRNA to pre-miRNA [31]. RNA interference of DROSHA resulted in accumulation of pri-miRNA and reduction of pre-miRNA and mature RNA [31]. Up to now, several groups have studied the role of *DROSHA* in cancer [32,33]. It has reported that *DROSHA* rs644236 TT genotype and rs7737174 AA genotype were associated with breast cancer risk in postmenopausal women [32]. *DROSHA* 3’UTR rs10719 is in strong linkage disequilibrium with rs644236 based on the one thousand Genomes data (r^2^ = 0.88) [33]. In the present association study, we found that *DROSHA* 3’UTR rs10719TC polymorphism was associated with the risk of bladder cancer. Stratified analysis demonstrated that rs10719TC/CC genotypes can significantly increase the risk of bladder cancer, especially among males and smokers. One possible explanation is that cigarette smoking has been established as the most important risk factor in the development of bladder cancer, which contains hundreds of chemicals, such as polycyclic aromatic hydrocarbons [34,35]. Thus, ever smokers may be prone to be cancer. Another reason might be that compared with female, the male people are more likely to expose to accumulated environmental risk factors involved in the etiology of bladder cancer such as cigarette smoking, occupational exposures (i.e. dyestuff manufacture, leather work). Additionally, we have the relatively small sample size of female people. Therefore, we can not detect the significant association in female people. Weng et al. also proposed that rs10719TC polymorphism was related with malignant peripheral nerve sheath tumor risk, which supported our findings [33]. Over expression of DROSHA was shown to effect cell proliferation and predicted poor prognosis in esophageal cancer [36], ovarian cancer [37], breast cancer [38], and cervical cancer [39]. Previous study had also revealed that over expression of DROSHA can promote cell proliferation and inhibit cell apoptosis in bladder cancer [24]. Therefore, we hypothesized that *DROSHA* 3’UTR rs10719C allele can increase the risk of bladder cancer, mainly through regulating the expression of DROSHA.

It has been proposed that some of the 3’UTR polymorphisms may be in the miRNA binding site or in the vicinity of binding site and may interfere with miRNA function leading to differential gene expression to affect the development of cancer [19,20]. Our luciferase reported gene assays indicated that *DROSHA* rs10719 T to C substitution disrupted a binding site for hsa-miR-27b, resulting the increased levels of *DROSHA* 3’UTR luciferase expression. Thus, the C allele is associated with an increased risk of bladder cancer, potentially through increased DROSHA expression, which was consistent with the previous finding [24]. Furthermore, no significant difference of the *DROSHA* mRNA expression level among different rs10719TC genotypes was observed in bladder tumor tissues using RT-PCR assay. These results suggest that the SNP does not affect mRNA expression, however, given that miRNA binding to mRNAs does not always lead to transcript cleavage, and sometimes it leads to translation repression, it is possible that the SNP leads to a change in DROSHA protein. However, we did not test this in our study, and therefore remains a possibility, but not proven. These data suggested that hsa-miR-27b may affect DROSHA expression by regulating protein translation. It was worth to note that our functional findings were in agreement with the results of a case-control study.

In the present study, hsa-miR-27b was firstly reported to regulate the expression of *DROSHA* in bladder cancer. Hsa-miR-27b had been studied widely, which had been identified to be correlation with many kinds of cancers, such as colorectal cancer [40], neuroblastoma [41]. Recently, hsa-miR-27b was found to inhibit colorectal tumor progression and angiogenesis, through targeting VEGFC [40]. Lee et al. indicated that hsa-miR-27b could act as a tumor suppressor to inhibit cell growth, tumor progression and inflammatory response by targeting PPARγ 3’UTR in neuroblastoma cells [41]. In the present study, our data proposed that hsa-miR-27b may affect DROSHA expression by regulating protein tranlation. Further functional assays should be conducted to reveal the exact mechanism and pathway in bladder cancer.

Some limitations should be proposed in the present study. First, due to the lack of the genitourinary related co-morbidity data information, we were not able to investigate the association between SNPs and co-morbidity adequately. Results need to be confirmed in larger studies with more detailed genitourinary related co-morbidity data information. Second, we predicted *DROSHA* 3’UTR-related miRNA through several website, and considered that the combination of multiple prediction approaches would greatly reduce the possibility of false positive. At last, hsa-miR-27a/b was chosen. In the present study, rs10719TC was associated with bladder cancer risk and it was adjacent to the hsa-miR-27a/b binding site in *DROSHA* 3’UTR. Therefore, we performed luciferase assay to explore whether rs10719 T to C substitution can affect the binding activity of hsa-miR-27a/b with *DROSHA* 3’UTR. However, we also found that rs72547276 SNP laid in a predicted *DROSHA* binding site for miR-27b. Unfortunately, rs72547276 was not eligible for SNP inclusion criteria (MAF< 5% in CHB). Although this SNP located in the binding site, we did not do more work on this SNP. In the future, we should study this SNP possibly functional effect.

In conclusion, we identified the risk allele of rs10719TC located in *DROSHA* 3’UTR and rs10719 T to C substitution can affect DROSHA protein expression by hsa-miR-27b target, which provided the possible mechanism in bladder cancer risk. Our study revealed a new insight into bladder carcinogenesis. Furthermore, the association and functional study are warranted to validate our findings.

## Supporting Information

File S1 **Supporting figure and tables**. Figure S1, Quantitative real-time PCR was used to measure levels of hsa-miR-27a/b expression and DROSHA mRNA expression. (A, B) The levels of hsa-miR-27a/b expression were measured in four cell lines. In (A), relative hsa-miR-27a expression level was higher in J82 cells than in others. In (B), relative hsa-miR-27b expression level was higher in J82 cells than in others. Fold changes are normalized to the expression levels of U6. (C) Association between rs10719TC and DROSHA mRNA expression in bladder cancer tissues. 61 bladder cancer tissues were used to assess the expression of DROSHA mRNA by Quantitative real-time PCR. The frequencies of TT, TC and CC genotypes were 32, 24 and 5, respectively. *P* value was calculated by non-parametric Kruskal-Wallis H test of three genotypic groups. The fold change was normalized against GAPDH. Table S1, Primer information of Real-time PCR. Table S2, Stratification analyses between DICER and DROSHA polymorphisms and risk of bladder cancer.
(DOC)
