# Supplementary material for: Genetic Variation in DROSHA 3’UTR Regulated by hsa-miR-27b Is Associated with Bladder Cancer Risk
Source: PLoS One. 2013 Nov 28;8(11):e81524. doi: 10.1371/journal.pone.0081524 (PMC3842954; doi:10.1371/journal.pone.0081524)

**Table S1.** Primer information of Real-time PCR

| Name | Forward Primer (5’-3’) | Reverse Primer (5’-3’) |
| --- | --- | --- |
| URP | TGGTGTCGTGGAGTCG |  |
| hsa-miR-27a | ACACTCCAGCTGGGTTCACAGTGGCTAAG | CTCAACTGGTGTCGTGGAGTCGGCAATTCAGTTGAGGCGGAACT |
| hsa-miR-27b | ACACTCCAGCTGGGTTCACAGTGGCTAAG | CTCAACTGGTGTCGTGGAGTCGGCAATTCAGTTGAGGCAGAACT |
| U6 | CTCGCTTCGGCAGCACA | AACGCTTCACGAATTTGCGT |
| *DROSHA* | ATGAGACGAGAAGTAACGGTGGAG | GCATTGGTGGTAGCGGATATGATG |
| *GAPDH* | GAAATCCCATCACCATCTTCCAGG | GAGCCCCAGCCTTCTCCATG |

URP: universal reverse primer

**Table S2**. Stratification analyses between *DICER* and *DROSHA* polymorphisms and risk of bladder cancer

| Genotypes | Cases/Controls | Sex (Cases/Controls) | |  | Smoking status (Cases/Controls) | |
| --- | --- | --- | --- | --- | --- | --- |
|  |  | male | female |  | Never | Ever |
| *DICER* |  |  |  |  |  |  |
| rs12323635C>T |  |  |  |  |  |  |
| CC | 266/286 | 219/234 | 47/52 |  | 125/167 | 141/119 |
| CT/TT | 414/424 | 332/339 | 82/85 |  | 175/268 | 239/156 |
| OR (95% CI)^a^ | 1.03 (0.83-1.28) | 1.03 (0.81-1.32) | 1.04 (0.63-1.73) |  | 0.87 (0.64-1.17) | 1.26 (0.91-1.73) |
| *P^a^* | 0.793 | 0.808 | 0.872 |  | 0.350 | 0.159 |
| rs13078T>A |  |  |  |  |  |  |
| TT | 603/640 | 490/515 | 113/125 |  | 265/398 | 338/242 |
| AT/AA | 76/83 | 62/67 | 14/16 |  | 34/47 | 42/36 |
| OR (95% CI)^a^ | 0.95 (0.68-1.33) | 0.95 (0.66-1.39) | 0.93 (0.43-2.04) |  | 1.09 (0.68-1.74) | 0.82 (0.51-1.32) |
| *P^a^* | 0.768 | 0.800 | 0.861 |  | 0.732 | 0.410 |
| rs1057035T>C |  |  |  |  |  |  |
| TT | 548/577 | 436/464 | 112/113 |  | 243/354 | 305/223 |
| TC/CC | 137/153 | 118/123 | 19/30 |  | 61/96 | 76/57 |
| OR (95% CI)^a^ | 0.96 (0.74-1.25) | 1.04 (0.78-1.39) | 0.67 (0.35-1.28) |  | 0.94 (0.66-1.35) | 0.97 (0.66-1.43) |
| *P^a^* | 0.753 | 0.793 | 0.228 |  | 0.735 | 0.878 |
| rs3742330A>G |  |  |  |  |  |  |
| AA | 302/331 | 242/263 | 60/68 |  | 143/196 | 159/135 |
| AG/GG | 381/396 | 311/321 | 70/75 |  | 159/253 | 222/143 |
| OR (95% CI)^a^ | 1.05 (0.84-1.30) | 1.05 (0.82-1.33) | 1.07 (0.66-1.74) |  | 0.87 (0.65-1.17) | 1.31 (0.96-1.80) |
| *P^a^* | 0.686 | 0.719 | 0.791 |  | 0.348 | 0.090 |
| *DROSHA* |  |  |  |  |  |  |
| rs2291109A>T |  |  |  |  |  |  |
| AA | 421/419 | 341/333 | 80/86 |  | 188/265 | 233/154 |
| AT/TT | 264/311 | 213/254 | 51/57 |  | 116/185 | 148/126 |
| OR (95% CI)^a^ | 0.83 (0.67-1.03) | 0.80 (0.63-1.02) | 0.98 (0.60-1.60) |  | 0.88 (0.65-1.19) | 0.78 (0.57-1.08) |
| *P^a^* | 0.098 | 0.073 | 0.927 |  | 0.411 | 0.130 |
| rs10719T>C |  |  |  |  |  |  |
| TT | 352/413 | 283/340 | 69/73 |  | 162/245 | 190/168 |
| TC/CC | 332/314 | 270/244 | 62/70 |  | 142/204 | 190/110 |
| OR (95% CI)^a^ | 1.25 (1.01-1.55) | 1.34 (1.05-1.70) | 0.96 (0.59-1.56) |  | 1.04 (0.78-1.40) | 1.56 (1.14-2.14) |
| *P^a^* | 0.041 | 0.018 | 0.879 |  | 0.780 | 0.006 |
| rs642321C>T |  |  |  |  |  |  |
| CC | 197/176 | 157/135 | 40/41 |  | 84/112 | 113/64 |
| CT/TT | 488/554 | 397/452 | 91/102 |  | 220/338 | 268/216 |
| OR (95% CI)^a^ | 0.79 (0.62-1.01) | 0.77 (0.58-1.01) | 0.88 (0.52-1.49) |  | 0.89 (0.64-1.24) | 0.68 (0.47-0.97) |
| *P^a^* | 0.056 | 0.055 | 0.622 |  | 0.477 | 0.034 |

^a^ Adjusted for age, sex, and smoking status (never, and ever) in logistic regression model.

**Figure S1.** Quantitative real-time PCR was used to measure levels of hsa-miR-27a/b expression and *DROSHA* mRNA expression. (A, B) The levels of hsa-miR-27a/b expression were measured in four cell lines. In (A), relative hsa-miR-27a expression level was higher in J82 cells than in others. In (B), relative hsa-miR-27b expression level was higher in J82 cells than in others. Fold changes are normalized to the expression levels of U6. (C) Association between rs10719T>C and *DROSHA* mRNA expression in bladder cancer tissues. 61 bladder cancer tissues were used to assess the expression of *DROSHA* mRNA by Quantitative real-time PCR. The frequencies of TT, TC and CC genotypes were 32, 24 and 5, respectively. *P* value was calculated by non-parametric Kruskal-Wallis H test of three genotypic groups. The fold change was normalized against *GAPDH*.

**Figure S1.**


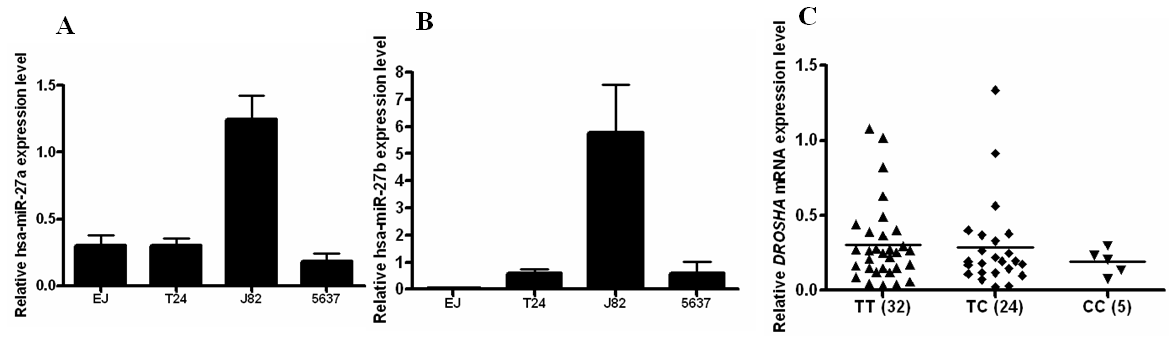

Supplement: File S1 — Supporting figure and tables. Figure S1, Quantitative real-time PCR was used to measure levels of hsa-miR-27a/b expression and DROSHA mRNA expression. (A, B) The levels of hsa-miR-27a/b expression were measured in four cell lines. In (A), relative hsa-miR-27a expression level was higher in J82 cells than in others. In (B), relative hsa-miR-27b expression level was higher in J82 cells than in others. Fold changes are normalized to the expression levels of U6. (C) Association between rs10719TC and DROSHA mRNA expression in bladder cancer tissues. 61 bladder cancer tissues were used to assess the expression of DROSHA mRNA by Quantitative real-time PCR. The frequencies of TT, TC and CC genotypes were 32, 24 and 5, respectively. P value was calculated by non-parametric Kruskal-Wallis H test of three genotypic groups. The fold change was normalized against GAPDH. Table S1, Primer information of Real-time PCR. Table S2, Stratification analyses between DICER and DROSHA polymorphisms and risk of bladder cancer. (DOC) [file pone.0081524.s001.doc]
